# Handheld Multispectral Fluorescence Imaging System to Detect and Disinfect Surface Contamination

**DOI:** 10.3390/s21217222

**Published:** 2021-10-30

**Authors:** Mitchell Sueker, Kristen Stromsodt, Hamed Taheri Gorji, Fartash Vasefi, Nadeem Khan, Taylor Schmit, Rangati Varma, Nicholas Mackinnon, Stanislav Sokolov, Alireza Akhbardeh, Bo Liang, Jianwei Qin, Diane E. Chan, Insuck Baek, Moon S. Kim, Kouhyar Tavakolian

**Affiliations:** 1 Biomedical Engineering Program, University of North Dakota, Grand Forks, ND 58202, USA; mitchell.sueker@und.edu (M.S.); kristen.stromsodt@und.edu (K.S.); hamed.taherigorji@und.edu (H.T.G.); bo.liang@und.edu (B.L.); 2 SafetySpect Inc., 4200 James Ray Dr., Grand Forks, ND 58202, USA; fvasefi@safetyspect.com (F.V.); nmackinnon@safetyspect.com (N.M.); ssokolov@safetyspect.com (S.S.); aakhbardeh@safetyspect.com (A.A.); 3 School of Medicine and Health Sciences, University of North Dakota, Grand Forks, ND 58202, USA; nadeem.khan@und.edu (N.K.); taylor.schmit@und.edu (T.S.); rangati.varma@ndus.edu (R.V.); 4 USDA/ARS Environmental Microbial and Food Safety Laboratory, Beltsville Agricultural Research Center, Beltsville, MD 20705, USA; jianwei.qin@usda.gov (J.Q.); diane.chan@usda.gov (D.E.C.); insuck.baek@usda.gov (I.B.); moon.kim@usda.gov (M.S.K.)

**Keywords:** fluorescence imaging, UV disinfection, saliva and respiratory droplets, cleanliness verification, sanitization documentation

## Abstract

Contamination inspection is an ongoing concern for food distributors, restaurant owners, caterers, and others who handle food. Food contamination must be prevented, and zero tolerance legal requirements and damage to the reputation of institutions or restaurants can be very costly. This paper introduces a new handheld fluorescence-based imaging system that can rapidly detect, disinfect, and document invisible organic residues and biofilms which may host pathogens. The contamination, sanitization inspection, and disinfection (CSI-D) system uses light at two fluorescence excitation wavelengths, ultraviolet C (UVC) at 275 nm and violet at 405 nm, for the detection of organic residues, including saliva and respiratory droplets. The 275 nm light is also utilized to disinfect pathogens commonly found within the contaminated residues. Efficacy testing of the neutralizing effects of the ultraviolet light was conducted for *Aspergillus fumigatus*, *Streptococcus pneumoniae*, and the influenza A virus (a fungus, a bacterium, and a virus, respectively, each commonly found in saliva and respiratory droplets). After the exposure to UVC light from the CSI-D, all three pathogens experienced deactivation (> 99.99%) in under ten seconds. Up to five-log reductions have also been shown within 10 s of UVC irradiation from the CSI-D system.

## 1. Introduction

Sanitation inspection is an ongoing concern for food distributors, restaurant owners, and others within the food industry. These individuals must prevent potential contamination and infection from spreading among workers and to consumers. The failure to meet legal requirements can result in the damage to institution or restaurant reputations, the loss of trust between the establishment and its workers and customers, and financial repercussions. All U.S. meat, poultry, and egg processing establishments are regulated by the Food Safety and Inspection Service (FSIS) and are required to have Sanitation Standard Operating Procedures (SSOPs). SSOPs are written procedures that the establishment develops and implements to prevent the direct contamination of food products. It is an establishment’s responsibility to execute the procedures as written in the SSOPs. Each establishment must maintain a daily documentation of the application and monitoring of the SSOPs, as well as any necessary corrective action. It is critically important to maintain the required documents as evidence of SSOP compliance and keep these up to date.

According to the Asia Pacific Society of Infection Control (APSIC) guidelines, “there are several methods for assessing environmental cleanliness: (1) a conventional program of direct and indirect observation (e.g., visual assessment, observation of performance, customer/staff satisfaction surveys); (2) an enhanced program of monitoring residual bioburden (e.g., environmental culture, adenosine triphosphate (ATP) bioluminescence); (3) and environmental marking tools (e.g., fluorescent dust marking of surfaces)” [1,2,3].

The visualization of fluorescence emission has great utility for food safety inspection. Food-related biological residues have been shown to have characteristic fluorescence emission spectra in visible (VIS) and near-infrared (NIR) wavelengths. Dairy cow feces show red fluorescence emissions peaking at 680 nm when excited by ultraviolet (UV) radiation (360 nm) [4,5]. Chlorophyll (Chl) in green plants has unique fluorescence emissions in the red and far-red regions, peaking at 685 nm and 730 nm [6,7]. Additionally, a number of plant constituents have been reported to have a UV emission at 340 nm and blue and green emissions peaking near 450 nm and 530 nm [6,7,8]. Meat products have been shown to have fluorescence emission in UV, blue, and green wavelengths. Proteins are known to emit UV fluorescence, and a variety of aromatic compounds emit fluorescence in blue and green wavelengths [8,9,10,11,12].

Multiple automatic imaging inspection techniques and systems have been used for food safety inspection. The online inspection of poultry carcasses for fecal contamination has been developed using a multispectral imaging system to visualize reflectance spectra features of visible wavelength regions [13,14]. A hyperspectral imaging system to detect fecal contamination on apples was developed, and this system can measure both reflectance and fluorescence in the visible to near-infrared [15,16]. A portable hyperspectral imaging system has also been developed to monitor sanitation procedures in food processing facilities. It showed the ability to detect minute quantities of juice from produce on food processing equipment [17,18]. An imaging device that is portable and capable of fluorescence-based contaminant detection on food products and food processing equipment can easily be integrated with workflows and sanitation audits in food handling facilities.

Some efforts have been made to commercialize line scan spectral imaging systems without disinfection capability, and with some documentation capabilities. Headwall Photonics, Inc. [19] has commercialized a line scan system after licensing a patent based on the research of one of our coauthors’ references cited in [15,16,17,18]. P&P Optica [20], Inc. has commercialized a similar line scan spectral imaging system that they claim is “able to detect, identify, and remove many types of foreign objects on production lines” as well as using “artificial intelligence to provide insights about shelf life, product composition, flavor, fat content, quality variation and much more”. VERITIDE Ltd. [21] is commercializing a fluorescence-based point measurement system to detect fecal contamination on meat carcasses, as well as a fluorescence-based production line imaging system for meat carcasses.

During the COVID-19 pandemic, many restaurants, dining facilities, kitchens, as well as food processing facilities, were heavily affected by virus contamination resulting in multiple facility shutdowns [22]. A need has emerged for new cleaning procedures and protocols to ensure all staff and customer high-touch areas are cleaned and monitored. Saliva and respiratory droplets can be one of the major contamination carriers for many diseases, including influenza, coronavirus, and Ebola [23,24]. Previous scientific studies directed to the detection of saliva and respiratory droplets have shown that short-wavelength UV excitation at 282 nm can be used to indicate the presence of saliva stains in which the α amylase enzyme gives off a characteristic emission spectrum at 345–355 nm, which can be identified using fluorescence imaging. The presence of the fluorescence emission at 345–355 nm with excitation at 282 nm proves to be a strong indicator of saliva, even when deposited on human skin [25].

In this paper, for the first time, we introduce a fast, convenient, and easy-to-use handheld system for “contamination, sanitization inspection, and disinfection (CSI-D)” that enables the rapid detection of saliva and respiratory droplets, and other organic residues that are present in kitchens, dining areas, and food processing facilities. The system provides the immediate disinfection and documentation of contaminants on surfaces that may cause disease spread. CSI-D can wirelessly communicate the inspection process, which allows remotely located personnel to immediately provide oversight and respond to inspection issues. The CSI-D system is not intended to be a primary disinfection or cleaning tool; instead, it acts as a post-cleaning audit solution complementary to other post-cleaning auditing tools (ATP, FT-IR, etc.), as well as providing documentation of cleanliness. The system’s disinfection capability is intended to provide spot disinfection only during audits or incident response and is not employed as a large area disinfection method (e.g., fogging).

The key innovations of this device encompass the visualization of contamination using fluorescence imaging, the disinfection of the contamination using UVC illumination, and the documentation of cleanliness. The combination of detection, disinfection, documentation, and verification is the core innovation from an operator’s point of view. Specific technical innovations include the ability to capture fluorescence images under bright ambient light situations in food processing facilities, institutional kitchens, and dining facilities. Previous systems (described above) had difficulties with bright ambient light and, often, could only function in darker rooms or under shrouding. Other innovations related to the UVC germicidal LEDs include the integration of safety systems based on sensors and software (LIDAR, gyroscope, motion detector, etc.) that help protect the operator and other personnel from accidental UVC light exposure. These sensors are also used to monitor motion and distance during the image capture process to ensure images are free from motion artifacts, such as image blur, and provide software-based guidance for operators when they are moving the camera too quickly or are too far away or too close. Finally, the image database and records of contamination for each location at each facility, combined with the local hazards and disease prevalence, will enable the future delivery of intelligent dynamic risk assessment associated with each surface to guide cleaning and inspection processes.

## 2. Materials and Methods

### 2.1. System Description

**Figure 1:**
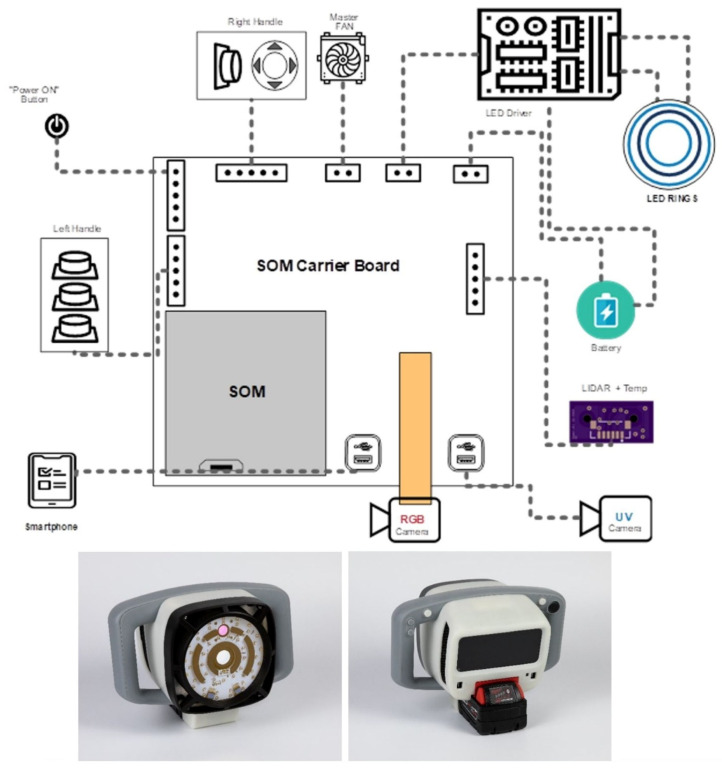
CSI-D system (top: system block diagram, bottom: CSI-D picture).

As shown in Figure 1, the CSI-D system consists of a handheld device that incorporates illumination, imaging, battery power, display, and processing units in a single system. The illumination module includes the 405 nm and 275 nm LED arrays, heat management, and driver circuits. The 275 nm LEDs were chosen because they were very close to the 282 nm excitation maximum wavelength of salivary amylase, were commercially available, and had high optical power. This wavelength is also a very effective germicidal wavelength. The 405 nm LEDs were selected because we previously used them for the detection of other organic residues, such as food residues containing fluorophores like collage; flavins; bacterial porphyrins; and chlorophyll. They are not used for the detection of saliva and respiratory droplets.

During the fluorescence imaging mode, the 405 nm or 275 nm LED arrays are turned on and off sequentially via electronic signals. During the disinfection mode, the 275 nm LEDs are turned on continuously for 2–5 s. The imaging system includes an RGB camera and a UV camera that communicate with the processing unit, which triggers the image acquisition and storage of fluorescence images of organic residues (RGB camera), or saliva and respiratory droplets and certain organic residues (UV camera) during fluorescence imaging. The RGB camera is also used in “ViewFinder” mode, whereby an operator can locate the area of interest to be scanned. The camera systems include lenses and spectral bandpass filters that select wavelengths specific to the contamination emission wavelength ranges.

The processing unit includes a system on module (SOM) board that controls the illumination and imaging modules to capture the fluorescence and background images under the appropriate illumination. The SOM processes images to provide meaningful information to the operator and for inspection records. The CSI-D system also includes a LIDAR module that communicates with the SOM module, which initializes and controls the LIDAR module and receives distance information from the rangefinder and temperature information from its temperature sensor. We are using an Android device as a smart display to provide an operator interface. The CSI-D system is designed to communicate with a dedicated cloud server in which all task lists are assembled, and inspection reports and video data are stored and managed.

The operator can select a disinfection mode using the hand controls and user interface. The system calculates how long the UVC illumination should be activated by calculating the surface distance using the LIDAR module.

### 2.2. Contamination Detection Algorithm

During the contamination detection procedure, we captured images that demonstrate the presence or absence of contamination on surfaces. Two fluorescence images were captured under blue/violet excitation using a color camera with a dual bandpass filter that passed selected green and red wavebands. We also captured an ultraviolet B (UVB) fluorescence image under UVC excitation using a UV-sensitive monochrome camera with a single bandpass filter. The image capture sequence comprised continuous image capture and background subtraction. For each fluorescence acquisition, we captured two frames: one frame with the LED illumination “ON” and one frame with the LED illumination “OFF.” The LED “ON” frame contained fluorescence emissions as well as any background light that could leak through the bandpass filter. The LED “OFF” frame contained only the background light. We implemented real-time background subtraction software to reduce the contribution of the background light to the final fluorescence image. The contamination detection algorithm was then applied to the background-subtracted fluorescence image frames. The UV camera image sensor was 2048 × 2048 pixels, and to increase the sensitivity and speed of the image capture, the pixels were binned to an image of 256 × 256 pixels. The RGB camera image sensor was 1280 × 960 pixels, but we selected a region of interest to read out that was 1024 × 768 pixels. At a distance of 20 cm, the field of view (FOV) of the RGB camera was 30 cm, and the FOV of the UV camera was 10.5 cm. To obtain reliable segmentation results for each image frame that are independent of fluorescence and background image intensity variations, due to the device movement during the inspection, the detection algorithm has to continuously adapt itself to changing fluorescence and background intensity levels. While imaging with a fixed-mount camera can allow simple conventional thresholding (e.g., Otsu’s method), we constantly moved the camera across different scenes, and therefore we adopted adaptive thresholding to change the threshold dynamically over each frame. This more sophisticated version of thresholding can accommodate the varying background and fluorescence conditions in each frame. Whenever intensities between an object’s fluorescence and the image background are significant, but their exact magnitude or location in the image is unknown, segmentation is possible by threshold optimization through the image intensity histogram. The image histogram represents the distribution and frequency of occurrence of the intensity for every pixel in the image. Because each image/frame varies in the frequency and distribution of these pixel intensities, the histogram can be normalized by the number of pixels for convenience of analysis.

During the first step of designing the algorithm, we generated a dataset from various contamination biofilms, such as olive oil, spinach juice, and egg white, on various surfaces, including steel, wood, and plastic. The ambient light is an important variable that needs to be considered in real-world applications of the device. We tried a wide range of ambient light from 0 to 90 foot-candle (FC) at intervals of 10 FCs, when capturing the images from each contamination on the different surfaces. We chose this range because the facilities we are targeting for CSI-D inspections can have ambient light up to 80 FCs.

**Figure 2:**
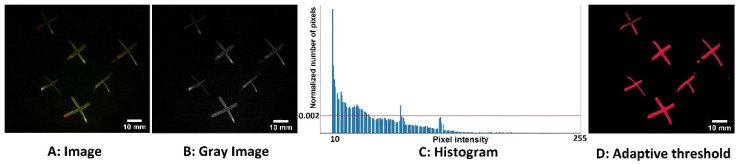
The adaptive threshold segmentation method used to detect fluorescence emission areas from olive oil residue marks. (**A**) The fluorescence image from the color camera; (**B**) the extracted green band monochrome image; (**C**) the histogram and threshold (red line); and (**D**) the resulting binary image mask. The scale bar for all images is set to 10 mm.

After capturing the images, we manually segmented the areas of the contamination. We then used a similarity measurement metric called “dice similarity” [26] and attempted to adjust the threshold level by minimizing the error between the manually segmented regions and the regions that resulted from the algorithm for the range of ambient light and surfaces. The method of thresholding we used was to create a histogram of a fluorescence image. We then normalized the histogram so that each bin was expressed as a percentage of the total number of pixels in the image. We then set a threshold: if the bin contained more than 0.2% of the pixels in the image, its pixel values were set to zero, and if the bin contained less than 0.2% of the pixels in the image but more than zero, its values were set to one. We selected 0.2% because it gave a good compromise between the detection of contaminant fluorescence and the detection of artifacts without applying additional spatial filters for size, etc. It also provided a good dice similarity (74.22 ± 0.32) to the manually segmented images. This is an interim segmentation method that is “good enough” for our present purposes, but we are working on applying more advanced machine learning-based segmentation. Figure 2 shows how we applied our adaptive threshold segmentation method to the green channel fluorescence with excitation 405 nm and a green emission band filtered to 510–560 nm. Specifically, Figure 2A demonstrates the color fluorescence image captured by the camera with both green and red bands, while Figure 2B is the extracted green band monochrome image. The histogram analysis described above is shown in Figure 2C, and Figure 2D displays the resulting binary image mask.

The segmentation algorithm has been implemented in the processing unit of the CSI-D system. After the color camera, fluorescence images were captured, and background intensity was subtracted; the red and green channels were separated to provide individual monochrome fluorescence images. Since the UV camera is a monochrome camera, color separation is not necessary. Currently, each channel is segmented separately to identify areas of potential contamination. In future work, we will investigate fluorescence color ratio analysis and other more sophisticated and unsupervised analyses that will use all three fluorescence signals to distinguish between different types of contamination and provide a better segmentation and classification of contamination.

### 2.3. Microbial/Viral Strain Preparation

We are using strains of bacteria, virus, and mold to test the effectiveness of disinfection by the CSI-D system. The experiments have been conducted in a biosafety level 2 laboratory, at the University of North Dakota School of Medicine & Health Sciences, with the following pathogens.

*Aspergillus fumigatus: A. fumigatus* [NIH 5233] was grown for 7 days at 37 °C on Sabouraud dextrose agar (SDA). The mature fungus was harvested using 0.1% Tween 80 in sterile phosphate-buffered saline (PBS), filtered through a sterile gauze, and resuspended in sterile PBS containing 0.1% Tween 80. The conidia were quantitated using a hemocytometer and resuspended at a final concentration of 5 × 10^6^/mL. The surface was decontaminated using 100% pure concentrated Cavicide and sterile Kimwipes, followed by 70% EtOH and sterile Kimwipes. After vortexing to mix, a 20 µL droplet of conidia was placed at the center of the surface for UVC light treatment (or no treatment for controls) using a P20 micropipette.

*Streptococcus pneumoniae: S. pneumoniae* serotype 6A strain BG7322 was provided by Rochester General Hospital Research Institute and was originally obtained from Sanofi Pasteur [27,28]. *S. pneumoniae* was resuspended to a final volume of 5.0 × 10^6^/mL or 2.5 × 10^6^/mL. The surface was decontaminated using 70% EtOH and sterile Kimwipes. After vortexing to mix, a 20 uL droplet was placed at the center of the surface using a P20 micropipette.

*Influenza A virus:* A mouse-adapted strain of influenza A virus strain A/PR/8/34 H1N1 (Charles River) was resuspended in an infection medium containing 1μg/mL TPCK-trypsin (Sigma) to obtain a virus concentration at 7 × 10^6^ PFU/μL (10^7^ TCID_50_/μL), where PFU is plaque forming units and TCID is the median tissue culture infectious dose. TCID_50_ was calculated using Equation (2), while PFU was calculated using Equation (3). The platform was disinfected using 70% EtOH and sterile Kimwipes. After vortexing to mix, a 20 μL droplet (containing 1.4 × 10^5^ PFUs, TCID_50_ 2 × 10^5^) was placed at the center of the platform using a P20 micropipette.

### 2.4. Disinfection Procedure

For the UVC irradiation by the CSI-D system illumination module, the specimen was placed anywhere within a 22.5 cm^2^ area that was marked on the surface of the platform. This area in the center of the illumination field has relatively uniform illumination power (>85%) from the UVC LEDs. Disinfection efficacy testing was performed by positioning the system at the designated distance from the contamination sample height, and manually pressing the two disinfection buttons to trigger the UVC illumination for the specified time duration. The illumination power density was measured with a UV radiometer (Opsytec Dr. Grobel GmBH) sensor, as the distances between the device and the sample were changed.

### 2.5. Post-Disinfection Treatment

After the disinfection treatment, the remaining pathogens were noted and used to calculate logarithmic reductions, as shown in Equations (4) and (5). The samples were observed and counted following the specified methods below.

*A. fumigatus:* For all samples exposed to UVC light or unexposed controls, a new sterile pipette tip was used to pipette the droplet and then resuspend it in 480 µL of sterile PBS. Samples were immediately placed on ice. For quantitation, the conidia were serially diluted 3 times at a ratio of 1:10 in sterile PBS and plated on fresh SDA. After incubation at 37 °C for 24 h, the colonies on the plate were counted, and the colony-forming units (CFU) were calculated using Equation (1).

*S. pneumoniae:* For all samples exposed to UVC light or unexposed controls, a new sterile pipette tip was used to pipette the droplet and then resuspend it in 480 µL of sterile PBS. Samples were immediately placed on ice. After UVC light exposure, the bacteria were serially diluted 3 times at a ratio of 1:10 in sterile PBS and plated in a volume of 10 µL on Trypticase soy agar II supplemented with 5% sheep’s blood (BD 221239). After incubation at 37 °C for 24 h, the colonies on the plate were counted, and the CFU was calculated using Equation (1).

*Influenza A virus:* For all samples exposed to UVC light or unexposed controls, the droplet was pipetted using a new sterile pipette tip and collected in 80 μL of infection medium in a fresh tube. After a quick vortex, the samples were kept on ice until further steps were taken. Test (UVC exposed) and control (not exposed) samples were subjected to TCID_50_ viral titration assay to determine the infectivity of the influenza A virus using Madin–Darby canine kidney (MDCK) cells. Each sample was 10-fold serially diluted and overlaid on a confluent monolayer of MDCK cells and then incubated at 37 °C in a CO_2_ incubator. On Day 5, the MDCK monolayers were fixed with 4% formaldehyde and stained with 0.5% crystal violet stain. The cells were observed for the presence or absence of cytopathic effect and scored as positive or negative events, respectively.

Colony-forming units (CFUs) were calculated using the following equation:

CFU = n × d × 25 (1)

where CFU is the number of colony-forming units in a 0.5 mL sample, n is equal to the number of countable colonies per 20 μL volume plated, and d is the dilution level yielding countable colonies.

TCID_50_/mL was calculated by:

(2) $${\mathrm{Log}}_{10}\left(\frac{{\mathrm{TCID}}_{50}}{\mathrm{ml}}\right)=x_{0}-\frac{d}{2}+\frac{d\times x_{i}}{n}+v$$

where x_0_ is the decimal logarithm of the initial dilution factor; d is the decimal logarithm of serial dilution factor; x_i_ is the score of positive events; n is the number of replicates; and v is the decimal logarithm of the reciprocal inoculation volume in mL.

Plaque forming units (PFUs)/mL were calculated using the following equation:

(3) $$\frac{PFU}{ml}=TCID_{50}\times 0.69$$

Logarithmic reductions for *A. fumigatus* and *S. pneumoniae* were calculated by:

(4) $$Log\;Reductions=log_{10}\left(\frac{CFU\;untreated}{CFU\;treated\;}\right)$$

Logarithmic reductions for the influenza A virus were calculated by:

(5) $$\;\;\;\;\;\;Log\;Reductions=log_{10}\left(\frac{TCID_{50}\;untreated}{TCID_{50}\;treated}\right)$$

Percent killing for *A. fumigatus* and *S. pneumoniae* were calculated by:

(6) $$\;\;\;\;\;\;\;\;\;\%\;Killing=100-\left(\frac{CFU\;treated}{CFU\;untreated}\times 100\right)$$

Percent killing for the influenza A virus was calculated by:

(7) $$\%\;Killing=100-\left(\frac{x_{i}\;treated}{x_{i}\;untreated}\times 100\right)$$

## 3. Results and Discussion

### 3.1. Contamination Detection

**Figure 3:**
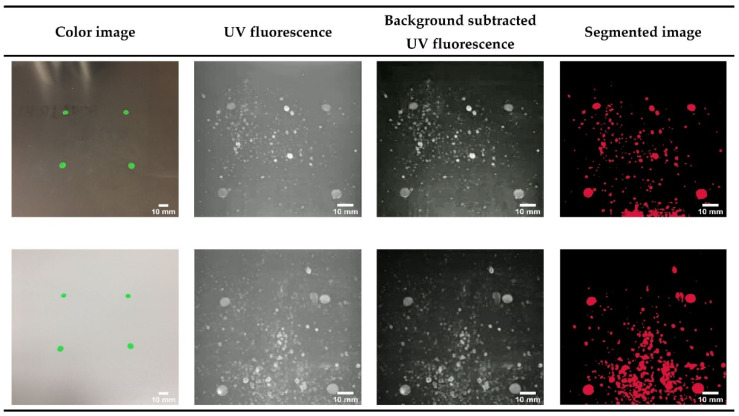
Images from the CSI-D system show saliva and respiratory droplets on stainless steel (top row) and plastic (bottom row). The smartphone color image column shows the green fluorescence paper dots affixed to the steel and plastic surfaces. The UV fluorescence column shows the images captured by the CSI-D UV camera. The background subtracted UV fluorescence column shows the UV image after background subtraction. The segmented image column shows the image segmentation mask after applying the adaptive thresholding algorithm. The fluorescent dots seen in the color image can be easily identified in the UV fluorescence image even with different fields of view. The scale bar for all images is set to 10 mm.

Figure 3 shows the image capture and background subtraction and segmentation of human saliva and respiratory droplets after stimulation of sneezing in a human volunteer. We have tested the system performance on stainless steel (top row) and plastic (bottom row) surfaces. We used four 6 mm diameter green fluorescence stickers (ChromaLabel) as location markers for the sneeze target area that were visible under conventional and UV wavelengths. The color images in the first column show each surface photographed using a conventional color camera. The UV fluorescence images are the images including the background contributions which can be from ambient light or a CMOS camera dark current. The background-subtracted UV fluorescence image shows the resulting image when the UV camera image with no LED illumination is subtracted from the UV fluorescence image. The last column shows the binary mask of the segmented image. The fluorescent stickers are used only for these experiments and will not be used for device operation in the field.

To determine the minimum detectable size for saliva and respiratory droplets, we sorted each segmented blob from the smallest to largest and considered the smallest 5% of these blobs. We determined the average area in pixels for the smallest blobs and then translated that to the real-world dimensions in millimeters. We found that the minimum diameter of the detected blobs was different for stainless steel (0.13 mm) versus a plastic surface (0.21 mm).

**Figure 4:**
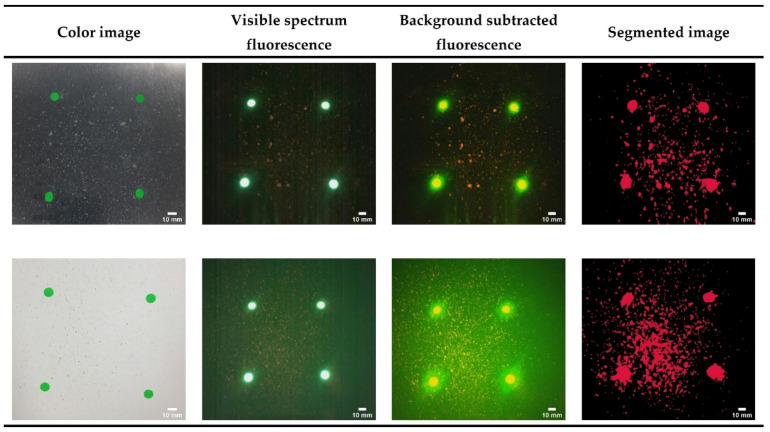
Images from the CSI-D system showing food residues (spinach) on stainless steel (top row) and plastic (bottom row) similar to those described in Figure 3, except the CSI-D RGB camera is used to capture visible light fluorescence instead of the CSI-D UV camera. In this series of images, the red band fluorescence is selected for adaptive thresholding to create the binary image segmentation mask. The scale bar for all images is set to 10 mm.

The raw image was converted to grayscale, and after the calculation of the histogram and normalization, the threshold level of 0.2% was applied to the image histogram. The result is shown in Figure 4. However, using the algorithm, some segmented regions were falsely identified as contaminated areas since the percentage of pixels of that intensity did not exceed 0.2%. The blobs with a pixel intensity that exceeded 0.2% of the pixels in the image were not selected as contaminations. It is worth mentioning that this imaging technology aims to identify if the surface is clean or unclean, and it is not intended as a quantitative assessment of biofilms.

Note that both the green and red fluorescence areas are shown together without any distinguishment.

Figure 4 shows image capture and the background subtraction and segmentation of food residues (spinach). We have tested the system performance on stainless steel (top row) and plastic (bottom row) surfaces. We, again, used four 6mm green fluorescence stickers as location markers. The color images in the first column show each surface photographed using a conventional color camera. The two visible spectrum fluorescence images are captured, and the background is subtracted, as described above, for the UV fluorescence images. Only the red and green channels from the RGB camera are used.

To isolate the red fluorescence signal, such as spinach or olive oil, we subtracted the red (R) channel from the green (G) channel and applied the adaptive thresholding algorithm. As can be seen in Figure 4, the red fluorescence areas were precisely distinguished by the algorithm. In the same way, to detect the green fluorescence (egg white/albumen), the G channel was subtracted from the R channel, and adaptive thresholding was applied.

**Figure 5:**
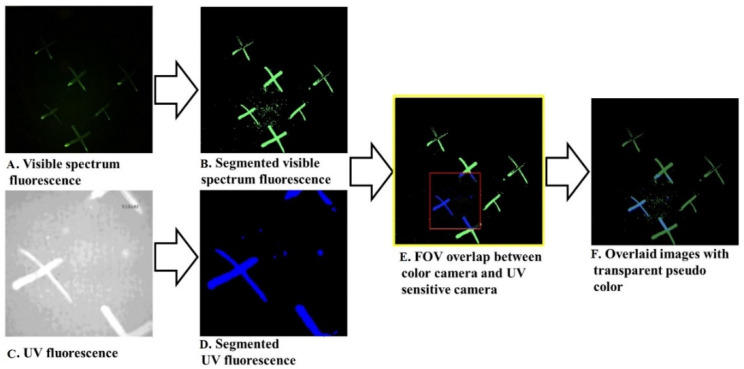
The image overlaying procedure for the registration of UV and RGB cameras with different fields of view. (**A**) The image from the color camera; (**B**) the segmented image from a color image; (**C**) the image from a UV camera; (**D**) the segmented image from a UV image; (**E**) a color camera FOV (yellow frame) versus a UV camera FOV (red frame); and (**F**) the result of the overlaying algorithm with 50% transparency. By cropping the image to the FOV of the UV camera, we can create a fully registered image for all three fluorescence emission bands.

Figure 5 shows the process of overlaying the images from the color and UV cameras. These cameras have different fields of view (FOV), image dimensions, and pixel sizes. The UV camera has a smaller FOV than the RGB camera. After resizing and rescaling, the UV images were translated relative to the RGB images, using a translation matrix to provide precise overlaying. Due to the fact that a translation magnitude is different at different distances, we captured images from a range of distances to determine the amount of shift required at each distance from the surface being image, along the x and y axes (t_x_, t_y_), and derived an equation from this for the translation matrix at any distance. The distance information is provided by the LIDAR range finder sensor.

(8) $$\mathrm{Translation}\;\mathrm{matrix}=\left[\begin{array}{cc} \;1\; & 0{\;t}_{x}\\ 0 & 1{\;t}_{y} \end{array}\right]$$

In Figure 5, the visible spectrum fluorescence areas are pseudo-colored with green, and the UV fluorescence areas are pseudo-colored with blue. To illustrate the accuracy of the overlaid contamination images, we set each channel at 50% transparency.

### 3.2. UVC Light Disinfection Results

**Figure 6:**
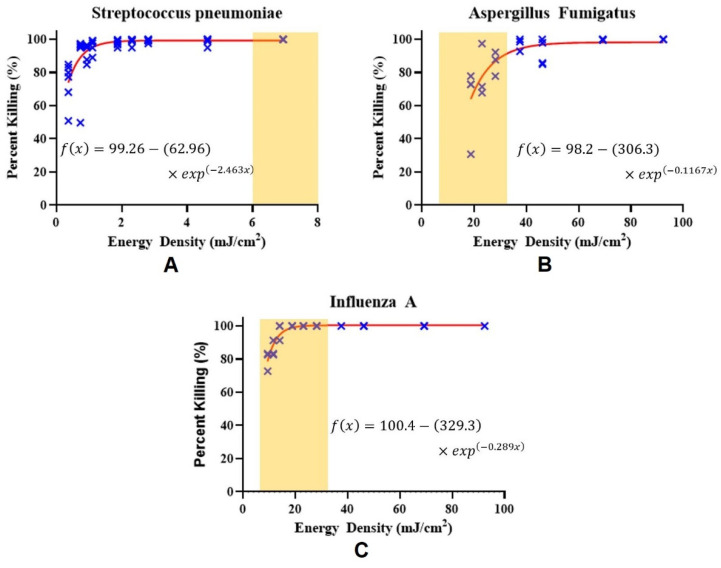
The percentage of bacteria killing rate as a function of UVC LED energy density. (**A**) *S. pneumoniae*, (**B**) *A. fumigatus*, and (**C**) *influenza A*. The yellow highlighted region shows the range of UVC light energy the device delivered to the surface, as described in detail in Section 3.2.

The disinfection results are shown in Figure 6. Experiments were repeated three times for each specific pairing of time and distance. The blue markers show the percentage of the pathogen killed relative to the control that did not receive UVC light exposure. The data were fitted to double exponential functions for each pathogen and are displayed as red lines in Figure 6. The yellow highlighted regions in Figure 6 show the UVC energy density that is delivered by the CSI-D device to the surface, ranging between 6–33 mJ/cm^2^. The system is designed to have a working distance between 12.5 cm to 25 cm. The UVC disinfection exposure time can be varied between 2–5 s. When the device is 25 cm away from the surface and the exposure time is 2 s, 6 mJ/cm^2^ of the UVC energy density will be delivered. When the exposure time has increased to 5 s, and the distance decreased to 12.5 cm, 33 mJ/cm^2^ of the UVC energy density will be delivered. The system has a rangefinder sensor that measures the distance in real-time and can calculate how much time is needed to complete pathogen deactivation at that distance. As shown in Figure 6A, UVC energy density above 6 mJ/cm^2^ deactivates the *S. pneumoniae* bacteria effectively.

As shown in Figure 6, bacteria required the least amount of UVC energy density to deactivate compared to viruses and fungi. This is consistent with other reports that show UVC light treatment to be extremely bactericidal [29,30]. However, UVC light treatment was able to reduce fungi and viruses below detectable levels at a higher UVC energy density. The higher UVC light doses required to deactivate the fungi were potentially due to the complex outer layer cell wall of the pathogens used [17].

Table 1 shows the verification of our results using the CSI-D system. Each data point indicates one sample of the specified pathogen that was exposed to the corresponding energy density of UVC light exposure.

**Figure 7:**
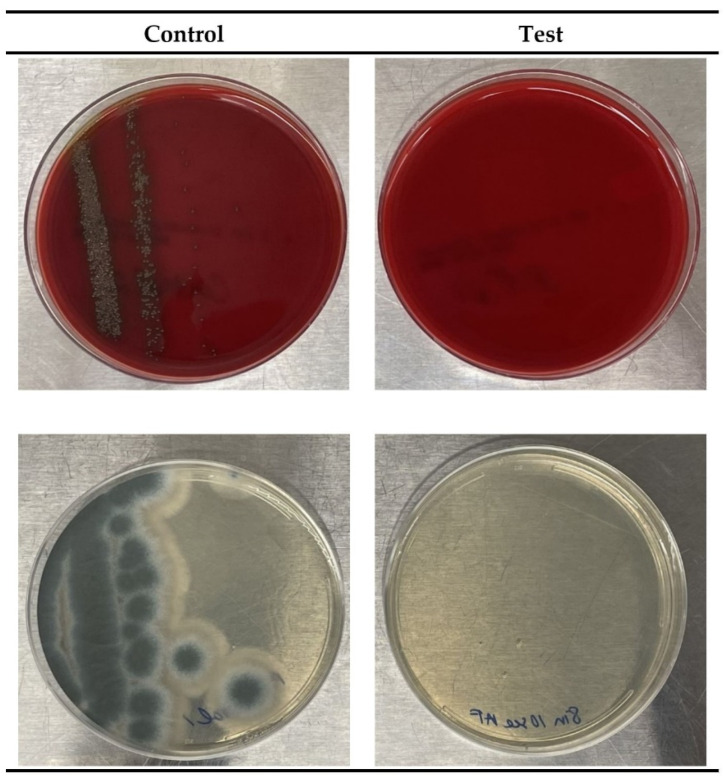
The UVC deactivation results for *S. pneumoniae* bacteria (top row) and *A. fumigatus* mold (bottom row) are shown by comparing an unexposed control (left column) and a UVC exposed plate (right column).

*A. fumigatus* was exposed to UVC irradiation at distances of 12.7 cm and 25.5 cm, and at exposure times of 5 s, 7 s, and 10 s. Only the 5 s and 7 s durations at 25.5 cm left any remaining biological contaminant, while the exposure times of 10 s at 20.3 cm and 5 s, 7 s, and 10 s at 25.5 cm, reduced the levels of *A. fumigatus* to undetectable. *S. pneumoniae* was tested at 25.5 cm and 38.1 cm distances, with the exposure times of 1 s, 2 s, and 3 s. The results of the *A. fumigatus* test (at 20.3 cm for 10 s) and the results of the *S. pneumoniae* test (at 38.1 cm for 1 s) are shown in Figure 7, below. All time durations at each distance resulted in > 99.99% killing of *S. pneumoniae*. Finally, the influenza A virus was tested at 12.7 cm and 20.3 cm, with the exposure time durations of 2 s, 3 s, 4 s, and 5 s. All time durations at each distance resulted in undetectable levels of the influenza A virus.

The percentage killing of the virus was determined by the established method of viral titer (TCID_50_) and cytopathic effect. In brief, the influenza A virus strain PR8 (H1N1) was resuspended in the infection medium at a concentration of 7 × 10^6^ PFU/μL. A 20 μL droplet (containing 1.4 × 10^5^ PFUs) was placed at the center of the platform. Post-UVC treatment (samples), or non-UVC treatment (controls), the droplet was collected in 80 μL of infection medium. For the TCID_50_ quantification, each sample was 10-fold serially diluted and overlaid on a confluent monolayer of MDCK cells and incubated at 37 °C in CO_2_ incubator. On day 5, cells were fixed with formaldehyde followed by crystal violet staining (see method for details). The cells were observed for the presence or absence of cytopathic effect (whitish plaques at the bottom of the well) and scored as a positive or negative event, respectively. For percent killing, the positive score of each sample was divided by the average positive score of the controls.

**Figure 8:**
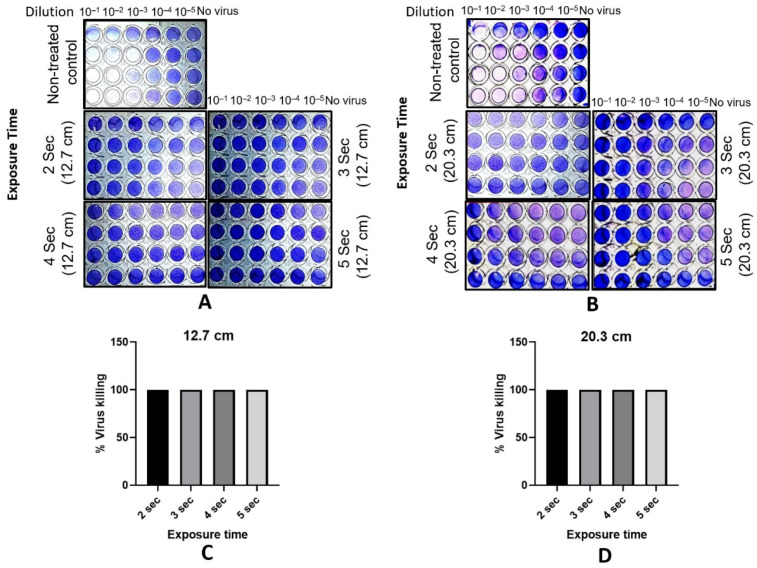
(**A**,**B**) The representative images of the quadrants of a 96-well plate showing the infectious activity of the influenza A virus on an MDCK cell monolayer. The non-treated quadrant (exposure time of 0 s) serves as a control. The clear wells in this quadrant indicate cell death due to the virus in the form of plaque development and the inability of the cell monolayer to take up the purple dye. The quadrants with the virus that were treated show no trace of cell death, indicating 100% killing, and are essentially identical to the column of wells in each quadrant with no virus. (**C**,**D**) The bar graphs show the percent killing of the virus (100%) after UVC light exposure times ranging from 2–5 s.

In Figure 8A,B, representative images are shown of 5 quadrants from 96-well plates presenting the infectivity of the influenza virus on the MDCK cell monolayer. Figure 8A shows the well plates that were exposed to UVC light at a distance of 12.7 cm from the device, and Figure 8B shows well plates exposed to UVC light at a distance of 20.3 cm. The untreated plate represents an exposure time of 0 s and serves as the control. Clear wells indicate cell death due to plaque formation caused by the virus at the various dilutions (10^−1^ to 10^−5^). Cell death and plaque formation result in the inability of the cell monolayer to take up the purple dye. In the no-virus control columns, MDCK cells were incubated with the infection medium only, and are representative of a cell monolayer with zero plaque formations due to the absence of the virus. Other panels show the deactivation of the virus treated with UVC light at various exposure times (2–5 s). Percent killing (deactivation) was calculated as described in the Methods Section above. Figure 8C,D show bar graphs representing the percent killing of the virus after varying exposures of UVC light. A virus killing of 100% was observed after each exposure time as, in the UVC-treated plate wells, the virus failed to cause cell death, even at the lowest dilution (only purple wells are observed for all exposure times and all dilutions except for the controls).

## 4. Conclusions

We have demonstrated a new handheld imaging system, CSI-D, which can rapidly detect, disinfect, and document possible organic residues which may host pathogens. The system uses light at two fluorescence excitation wavelengths (ultraviolet C at 275 nm and violet at 405 nm) for the detection of organic residues, including saliva and respiratory droplets. The 275 nm light is also used to disinfect pathogens commonly found in contamination residues.

We have demonstrated that the system can capture and segment fluorescence images of saliva and respiratory droplets, as well as organic residues, such as vegetable residue (spinach); fats (olive oil); and proteins (egg albumen), all under ambient room lighting conditions.

We have demonstrated that the UVC light in the system can deactivate bacterial, viral, and mold pathogens using *A. fumigatus*, *S. pneumoniae*, and the influenza A virus (a fungus, a bacterium, and a virus, respectively, which are each commonly found in saliva and respiratory droplets). After the exposure to UVC light from the CSI-D, all three pathogens experienced 100% sterilization or deactivation in under 10 seconds. Up to 5-log reductions (99.999%) have also been shown within 10 s of UVC irradiation from the CSI-D system.

When used as an auditing tool to verify and document cleanliness following sanitization, the CSI-D system can improve safety for food processing, preparation, and serving facilities and protect their staff and customers.

## Figures and Tables

**Table 1:** CSI-D irradiation results.

Pathogens	mJ/cm^2^	Minimum Log Reductions
*A. fumigatus*	26.23	1.47
	31.05	4.28
	36.11 47.61 56.05 66.73	2.58 4.28 4.28 4.28
*S. pneumoniae*	1.21	4.90
	2.32 3.63 5.14 7.27 10.10	4.90 4.90 4.90 4.90 4.90
*Influenza A virus*	9.40	5.25
	12.61	4.75
	14.18 19.27 19.36 25.27 26.32 33.31	5.25 5.25 4.75 5.25 4.75 4.75

## Data Availability

The data presented in this study are available on request from the corresponding author, subject to a confidentiality agreement. The data are not publicly available due to confidentiality agreements between the authors’ institutions and some of the measurement sites.
